# Investigating the association between serum ADAM/ADAMTS levels and bone mineral density by mendelian randomization study

**DOI:** 10.1186/s12864-023-09449-4

**Published:** 2023-07-19

**Authors:** Xin Lv, Yuhong Lin, Zhilei Zhang, Bo Li, Ziliang Zeng, Xu Jiang, Qiancheng Zhao, Wenpeng Li, Zheyu Wang, Canchun Yang, Haolin Yan, Qiwei Wang, Renyuan Huang, Xumin Hu, Liangbin Gao

**Affiliations:** 1 Department of Orthopedics, Sun Yat-sen Memorial Hospital of Sun Yat-sen University, 107 Yanjiang West Road, Guangzhou, 510120 China; 2 Faculty of Forensic Medicine, Zhongshan School of Medicine, Sun Yat-Sen University, Guangzhou, China

**Keywords:** Bone mineral density, ADAMTS5, Mendelian randomization, Quantitative heel ultrasound, Genome-wide association data, Genetic causality

## Abstract

**Purpose:**

A Disintegrin and Metalloproteinase (ADAM) and A Disintegrin and Metalloproteinase with Thrombospondin Motif (ADAMTS) have been reported potentially involved in bone metabolism and related to bone mineral density. This Mendelian Randomization (MR) analysis was performed to determine whether there are causal associations of serum ADAM/ADAMTS with BMD in rid of confounders.

**Methods:**

The genome-wide summary statistics of four site-specific BMD measurements were obtained from studies in individuals of European ancestry, including forearm (n = 8,143), femoral neck (n = 32,735), lumbar spine (n = 28,498) and heel (n = 426,824). The genetic instrumental variables for circulating levels of ADAM12, ADAM19, ADAM23, ADAMTS5 and ADAMTS6 were retrieved from the latest genome-wide association study of European ancestry (n = 5336 ~ 5367). The estimated causal effect was given by the Wald ratio for each variant, the inverse-variance weighted model was used as the primary approach to combine estimates from multiple instruments, and sensitivity analyses were conducted to assess the robustness of MR results. The Bonferroni-corrected significance was set at *P* < 0.0025 to account for multiple testing, and a lenient threshold *P* < 0.05 was considered to suggest a causal relationship.

**Results:**

The causal effects of genetically predicted serum ADAM/ADAMTS levels on BMD measurements at forearm, femoral neck and lumbar spine were not statistically supported by MR analyses. Although causal effect of ADAMTS5 on heel BMD given by the primary MR analysis (β = -0.006, -0.010 to 0.002, *P* = 0.004) failed to reach Bonferroni-corrected significance, additional MR approaches and sensitivity analyses indicated a robust causal relationship.

**Conclusion:**

Our study provided suggestive evidence for the causal effect of higher serum levels of ADAMTS5 on decreased heel BMD, while there was no supportive evidence for the associations of ADAM12, ADAM19, ADAM23, and ADAMTS6 with BMD at forearm, femoral neck and lumbar spine in Europeans.

**Supplementary Information:**

The online version contains supplementary material available at 10.1186/s12864-023-09449-4.

## Introduction

Decreased bone mineral density (BMD), the clinical sign of bone loss, is a critical risk factor of osteoporosis [1]. Osteoporosis is an aging-related degenerative disease characterized by reduction of bone mass and destruction of bone structure. Osteoporosis results in a decrease in bone strength and an increase in the risk of fracture, and brings a great challenge for the aging society due to its serious negative impact on the general health and life quality of postmenopausal women and the elderly [2]. BMD results examined by dual-energy X-ray absorptiometry (DXA)-scanning at forearm (FA-BMD), femoral neck (FN-BMD) and lumbar spine (LS-BMD) are commonly used in clinical settings, while recently, the estimated BMD (eBMD) measured by quantitative heel ultrasound has also been identified a highly heritable predictor of fracture [3]. Although a large number of risk factors and mechanic pathways of osteoporosis have been recognized by analyzing its genetic characteristics and clinical measurements [4], illuminating the complex pathogenesis of the changes in BMD is still a challenge for both clinicians and researchers.

The crucial metalloproteinases in the destruction of extracellular matrix proteins, A Disintegrin and Metalloproteinase (ADAM) and A-Disintegrin and Metalloproteinase with Thrombospondin Motif (ADAMTS), have been found involved in several bone diseases and related to biological process of bone and cartilage in different ways. In TIMP-3-deficient mouse models, the inhibition of ADAM and ADAMTS is partially relieved, and higher bone remodeling characterized by altered cortical and mineralization of trabecular bone and increased compositional heterogeneity can be observed at the same time [5]. At the late stage during differentiation of mesenchymal stem cells towards chondrocytes, the increased expression levels of ADAM19, ADAM23 and ADAMTS5 suggests a potential to regulate the phenotype, maturation and function of chondrocyte and a critical role in growth-plate organization and endochondral ossification [6]. Additionally, ADAMTS5 is regulated by Wnt/β-catenin signal, which is a critical developmental regulation pathway, and functions during the cartilage callus remodeling into bone during osseointegration [7, 8]. ADAM12 has been considered as an osteoclastic gene to promote osteoclastic resorption and bone loss [9–11]. POFUT2 has been identified impotant in bone development, while ADAMTS6 acts as a substrate of POFUT2 and significantly reduces during adipose derived stem cells undergoing osteogenesis [12, 13]. Such findings encourage us to further explore the potential of ADAM and ADAMTS in bone formation, development and remodeling, and the possibility as candidate biomarkers of bone loss and osteoporosis.

**Fig. 1:**
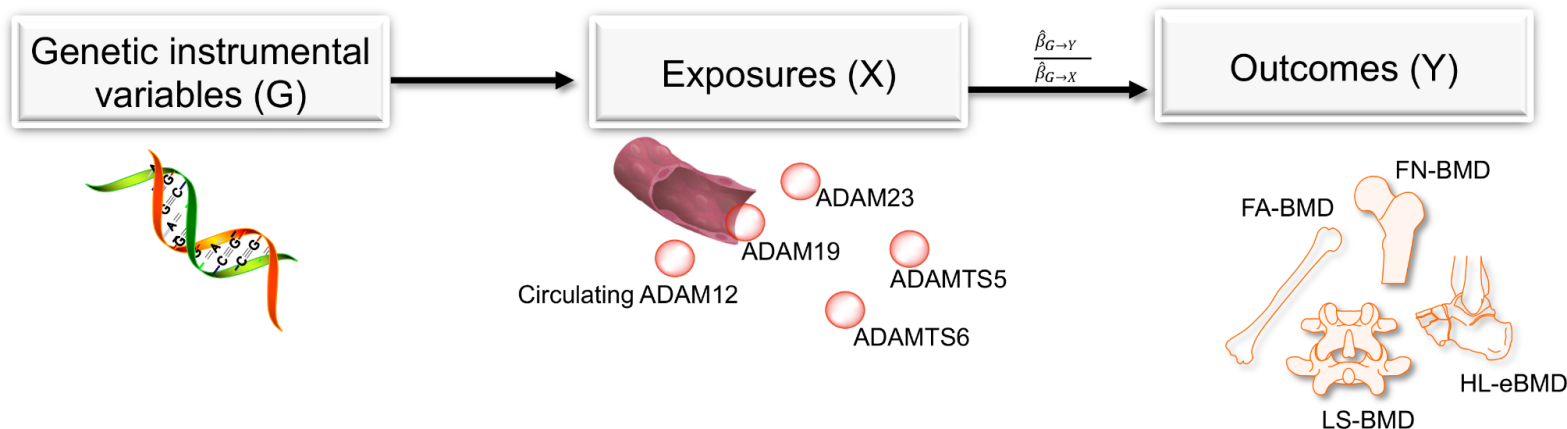
Schematic of the Mendelian randomization study investigating causal effects of metalloproteinases on bone mineral density. ADAM, A Disintegrin and Metalloproteinase; ADAMTS, A Disintegrin and Metalloproteinase with Thrombospondin Motif; BMD, bone mineral density; FA, forearm; FN, femoral neck; HL, heel; LS, lumbar spine; SNP, single nucleotide polymorphism

So far, the relationship between ADAM/ADAMTS and BMD remains inconclusive. Randomized controlled trials (RCT) are considered a gold standard when exploring causality, but the high time and money cost brings limitations for such studies. With more and more genetic data for complex diseases and traits turning publicly available, Mendelian Randomization (MR) analysis becomes an alternative approach to investigate the causal inference between exposures and outcomes [14]. The MR method employs single nucleotide polymorphism (SNPs) as instrumental variables, explains the causal relationship in a genetic view, and could draw conclusions that are similarly effective compared with RCT studies [15]. Besides, by using genome-wide association study (GWAS) summary statistics that are randomly allocated before birth and fixed at conception, MR is superior in avoiding confounders, which is the main cause of reverse causality and false-positive result in observational studies [16, 17]. Since GWAS has demonstrated that BMD is a highly polygenically heritable trait [18], several MR studies have been conducted and have recognized the causal associations of some biomarkers, behaviors and diseases with BMD such as serum estradiol concentrations, smoking, and type 2 diabetes, while some previously reported factors were not supported such as C-reactive protein and 25‑hydroxyvitamin D concentrations [19]. In this study, we performed a two-sample MR analysis (Fig. 1) to find out whether there is a genetic correlation between ADAM/ADAMTS and BMD. Our findings may provide genetically informative evidence for studies on new biomarkers and further understandings on the pathogenesis of osteoporosis.

## Materials and methods

The MR study was based on publicly accessible data. Informed consent and ethical approval have been obtained in original studies (Supplementary Table 1). We are particularly interested in five ADAM/ADAMTS metallopeptidases (ADAM12, ADAM19, ADAM23, ADAMTS5 and ADAMTS6), which were likely to exert influences on BMD as indicated in previous studies.

### Data sources of ADAM/ADAMTS metallopeptidases

We retrieved summary-level association data for ADAM/ADAMTS from a recent GWAS [20] conducted in European participants (n = 5336 ~ 5367). Serum concentrations of ADAM/ADAMTS were measured by the SOMAscan platform (SomaLogic Inc, Boulder, United States). Individuals were genotyped with Illumina array, and 7,506,463 variants with minor allele frequency > 1%, Hardy-Weinberg equilibrium *P* > 1 × 10^− 6^, call rate > 95% and imputation score > 0.7 were kept. Association tests were conducted for each unique protein adjusted for age, sex, five principal components, and genotyping platform. Stepwise conditional association analysis was also performed to give independent lead variants. We obtained SNPs associated with ADAM12, ADAM19, ADAM23, ADAMTS5 and ADAMTS6 reaching genome-wide significance (*P* < 5 × 10^− 8^) as genetic instrumental variables for the ensuing MR analyses.

### Summary statistics of bone mineral density

Summary statistics for BMD used in this study were obtained from the datasets of the largest GWAS on BMD so far released by GEnetic Factors for OSteoporosis Consortium and the UK Biobank. The DXA-derived BMD associated SNPs were extracted from the large-scale meta-analysis performed by Zheng et al. [21] in 2015, including FA-BMD (n = 8,143), FN-BMD (n = 32,735) and LS-BMD (n = 28,498) in individuals of European ancestry from the general population. The eBMD associated SNPs were gained from the study performed by Morris, et al. [22] in 2018, which assessed genetic determinants of BMD as estimated by heel quantitative ultrasound in Europeans (n = 426,824). Effect size represented changes in the unit of standard deviation (SD) of BMD per additional copy of effect allele. For instrumental SNPs which were not present in the datasets of BMD, variant proxies (*r*^*2*^ ≥ 0.8) were adopted if available. Summary statistics of ADAM/ADAMTS metallopeptidases and site-specific BMD were harmonized in terms of the effect allele and alternate allele of each SNP. The merged datasets (Supplementary Tables 2–5) were used in subsequent MR analyses.

### Mendelian randomization

We used the R software, version 3.6.3 (R Foundation for Statistical Computing, Vienna, Austria) and the *TwoSampleMR* package, version 0.5.6 [23] to perform the MR analysis (Supplementary Text). Given the effect of SNP_k_ on the serum level of ADAM/ADAMTS (${\widehat{\beta }}_{{X}_{k}}$, ${\widehat{\sigma }}_{{X}_{k}}$) and its effect on BMD (${\widehat{\beta }}_{{Y}_{k}}$, ${\widehat{\sigma }}_{{Y}_{k}})$ per additional copy of effect allele (n = 0, 1, 2) from the GWAS, causal effect of ADAM/ADAMTS on BMD ($\text{S}\text{N}{\text{P}}_{k}\to {X}_{k}\to {\text{Y}}_{k}$) can be estimated using the Wald ratio [24], namely with its associated standard error given by.

1 $${\widehat{\theta }}_{k}= \frac{{\widehat{\beta }}_{{Y}_{k}}}{{\widehat{\beta }}_{{X}_{k}}}$$

2 $$se\left({\widehat{\theta }}_{k}\right)=\sqrt{var\left({\widehat{\theta }}_{k}\right)}= \frac{{\widehat{\sigma }}_{{Y}_{k}}}{{\widehat{\beta }}_{{X}_{k}}}$$

The inverse variance weighted (IVW) model was adopted as the primary MR approach to compute overall estimates from multiple instrumental variables [25]. Causal effect size ${\widehat{\theta }}_{IVW}$ and standard error ${\widehat{\sigma }}_{IVW}$ were derived as below.

3 $${\widehat{\theta }}_{IVW}=\frac{{{\Sigma }}_{k}{\widehat{\beta }}_{{X}_{k}}{\widehat{\beta }}_{{Y}_{k}}{\widehat{\sigma }}_{{Y}_{k}}^{-2}}{{{\Sigma }}_{k}{\widehat{\beta }}_{{X}_{k}}^{ 2}{\widehat{\sigma }}_{{Y}_{k}}^{-2}}$$

4 $${\widehat{\sigma }}_{IVW}=\frac{1}{\sqrt{{{\Sigma }}_{k}{\widehat{\beta }}_{{X}_{k}}^{ 2}{\widehat{\sigma }}_{{Y}_{k}}^{-2}}}$$

Estimated effect size from the IVW method would be biased when invalid instrumental SNPs with unbalanced horizontal pleiotropy were present. Hence, two additional models, weighted median and MR-Egger were adopted. Weighted median estimator was robust when more than 50% SNPs were valid instruments [26]. MR-Egger regression intercept was capable of identifying unbalanced horizontal pleiotropy, whereas the regression slope would give an causal estimate adjusting for pleiotropy [27]. Weighted median and MR-Egger approaches were considered as sensitivity analyses tools, which tend to give less precise point estimates accompanied by wide confidence intervals (CI). As complementary tools, we also examined potential outlier variants and their heterogenous effect using the scatter, funnel and leave-one-out plot. Causal effect estimates were interpreted as BMD changes in the unit of SD with elevated levels of ADAM/ADAMTS. Statistical significance was set at *P* < 0.05/(5*4) = 0.0025 using the Bonferroni correction, and *P* < 0.05 was deemed as suggestive evidence for a causal relationship. Finally, mRnd1, a well-established tool for a posteriori power estimation in Mendelian randomization analyses [28], was used for power calculation.

## Results

### Genetically-predicted ADAM/ADAMTS levels in relation to FA-, FN- and LS-BMD

**Fig. 2:**
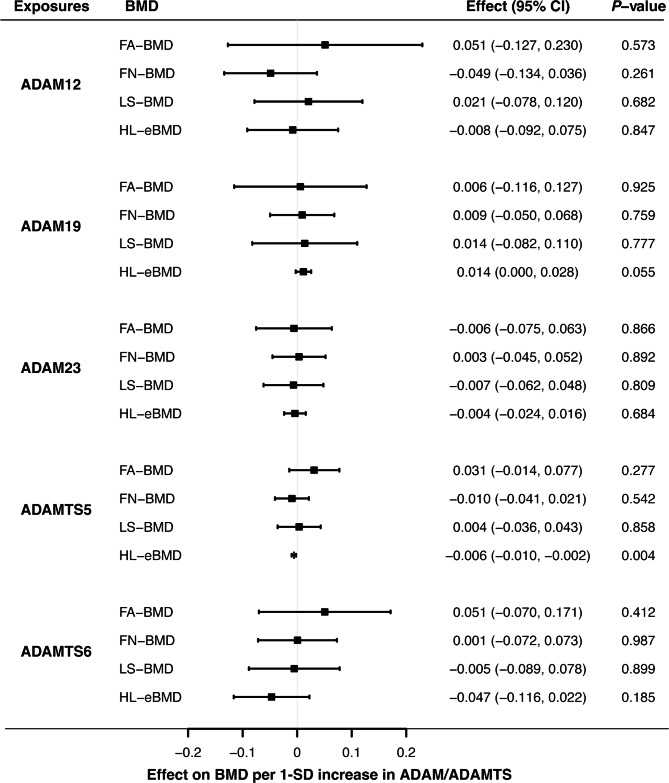
Effects of ADAM/ADAMTS metalloproteinases on bone mineral density estimated by the Mendelian randomization analyses. Causal estimates given by the inverse weighted variance model were delineated with forest plots, with solid blocks representing effect sizes and horizontal lines meaning confidence intervals. ADAM, A Disintegrin and Metalloproteinase; ADAMTS, A Disintegrin and Metalloproteinase with Thrombospondin Motif; BMD, bone mineral density; CI; confidence interval; eBMD, bone mineral density estimated by quantitative ultrasound; FA, forearm; FN, femoral neck; HL, heel; LS, lumbar spine; SD, standard deviation

MR analyses suggested that serum concentrations of ADAM12, ADAM19, ADAM23, ADAMTS5 and ADAMTS6 were not associated with FA-, FN- and LS-BMD. According to the primary MR method (Fig. 2), causal effects of each metallopeptidase on FA-BMD were 0.051 (-0.127 to 0.230, *P* = 0.573) per 1-unit increment in ADAM12 levels, 0.006 (-0.116 to 0.127, *P* = 0.925) per 1-unit increment in ADAM19 levels, -0.006 (-0.075 to 0.063, *P* = 0.866) per 1-unit increment in ADAM23 levels, 0.031 (-0.014 to 0.077, *P* = 0.277) per 1-unit increment in ADAMTS5 levels, and 0.051 (-0.070 to 0.171, *P* = 0.412) per 1-unit increment in ADAMTS5 levels. Likewise, genetically-predicted serum levels of ADAM12, ADAM19, ADAM23, ADAMTS5 and ADAMTS6 were not in association with FN-BMD or LS-BMD (Supplementary Tables 6–8).

### Serum ADAMTS5 concentrations were associated with heel eBMD

**Fig. 3:**
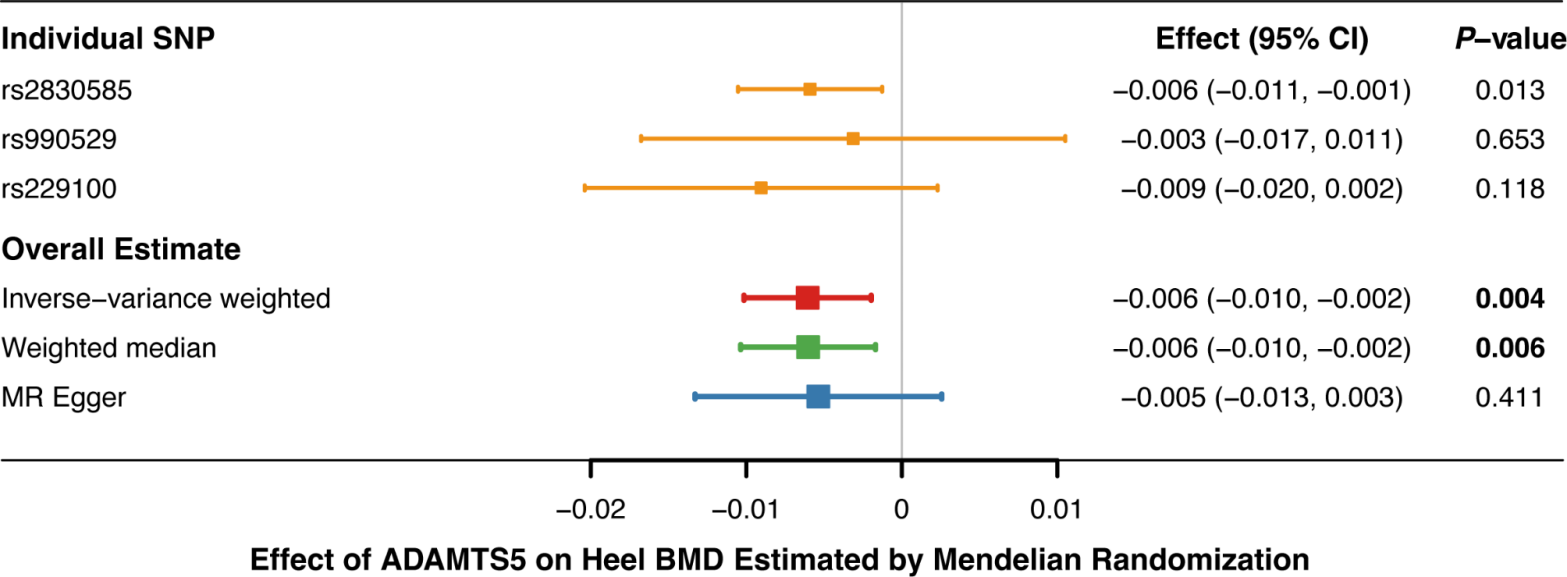
Association of genetically-predicted serum ADAMTS5 levels with heel bone mineral density. Effect estimates derived from individual SNP were given by Wald ratios, and overall estimates were computed using three MR approaches, among which the primary method—inverse weighted variance MR suggested that elevated concentrations of ADAMTS5 were associated with decreased heel BMD (β = -0.006, *P* = 0.004). ADAMTS5, A Disintegrin and Metalloproteinase with Thrombospondin Motif 5; BMD, bone mineral density; CI; confidence interval; MR, Mendelian randomization; SNP, single nucleotide polymorphism

**Fig. 4:**
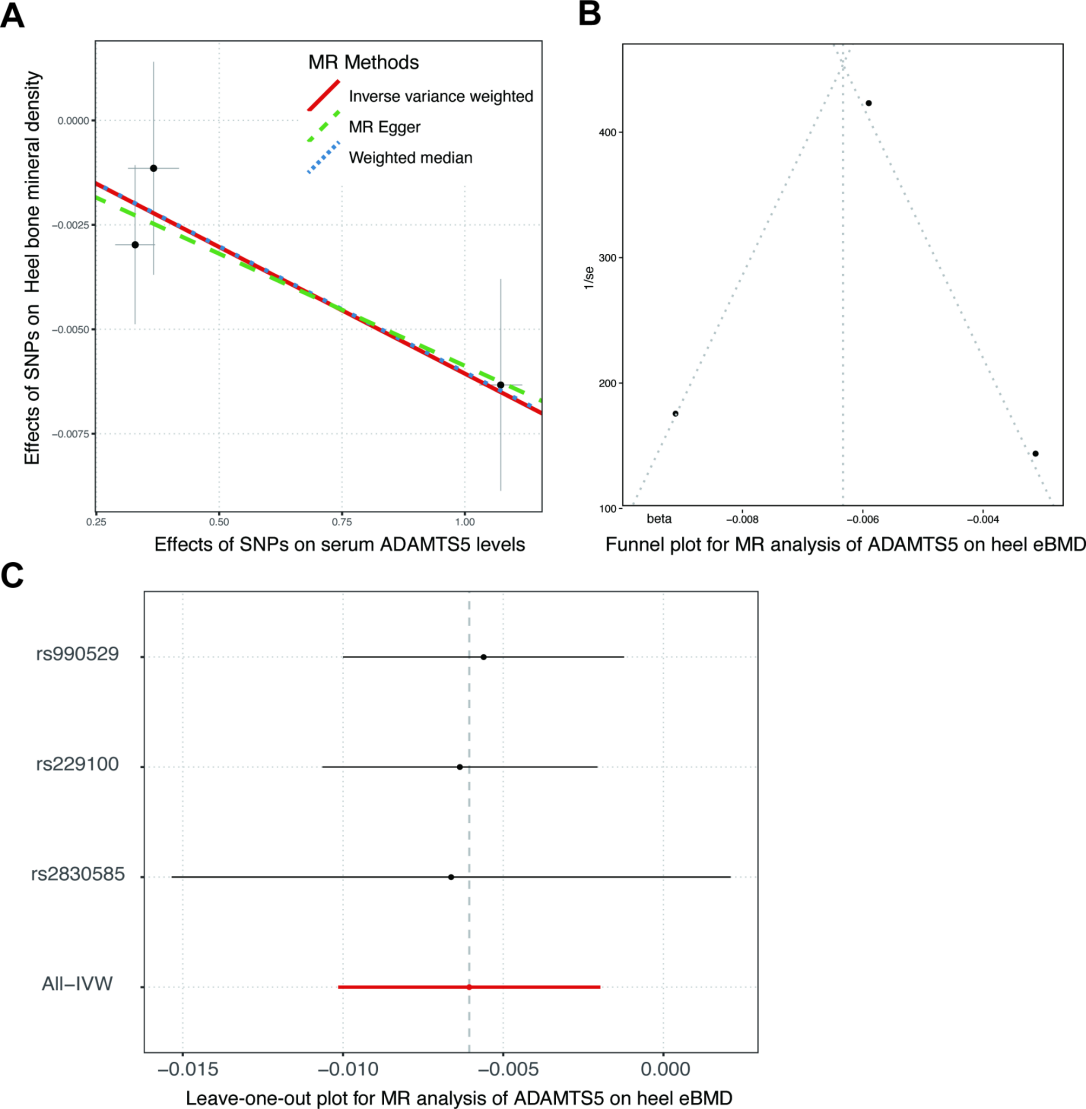
Sensitivity analyses for the Mendelian randomization of ADAMTS5 on heel bone mineral density. In the scatter pot **(A)**, each instrumental SNP was presented with a black point, its associated vertical gray line (effect on heel BMD) and the crossed horizontal gray line (effect on serum ADAMTS5). Colored lines illustrated overall causal estimates given by three MR methods, inverse-variance weighted (red solid), weighted median (blue dotted) and MR-Egger (green dashed). For each SNP, the causal effect against its inverse standard error were depicted in the funnel plot (**B**). Causal estimates from all instrumental variables except for the removal one in turn were delineated in the leave-one-out plot (**C)**. On the whole, no evident outlier was detected in the sensitivity analyses, which would otherwise heterogeneously or disproportionately influence the overall estimate. ADAMTS5, A Disintegrin and Metalloproteinase with Thrombospondin Motif 5; BMD, bone mineral density; MR, Mendelian randomization; SNP, single nucleotide polymorphism

Overall, MR analyses showed that elevated concentrations of ADAMTS5 were associated with decreased heel eBMD, but did not support causal effects of ADAM12, ADAM19, ADAM23 and ADAMTS6 (Fig. 2, Supplementary Tables 9–10). Causal relationships between ADAMTS5 and heel eBMD were suggested by the IVW (*P* = 0.004) and weighted median method (*P* = 0.004), albeit not reaching Bonferroni-corrected significance. The causal effect estimate derived from the MR-Egger slope, -0.005 (-0.013 to 0.003, *P* = 0.411) was directionally consistent, yet less precise (Fig. 3). MR-Egger intercept indicated no unbalanced horizontal pleiotropy (Intercept = -0.001, *P* = 0.874). Individual causal estimate given by the Wald ratio from three instrumental SNPs was examined, and overall causal estimate showed no evident heterogeneity through sensitivity analyses (Fig. 4). On the whole, the MR analysis provided suggestive evidence (0.0025 < *P* < 0.05) for the causal effect of ADAMTS5 on heel eBMD.

## Discussion

ADAM and ADAMTS are two large metalloproteinase families with important roles in numerous physiological processes, especially in regulating cell adhesion, migration, proteolysis and signaling [29, 30]. Previous studies have discovered ADAM/ADAMTS family proteins were in relation with neurodegenerative disorders, airway dysfunctions, cancers, atherosclerosis and many other diseases [31–34], but little is known about the correlation between ADAM/ADAMTS metalloproteinase and osteoporosis. GWASs report summary data associated with a trait for the strongest SNP in a certain genomic region and typically enroll many regions across the entire genome, which enables researchers to analyze a huge number of uncorrelated genetic variants [35]. Therefore, we used MR method, an attractive tool for estimating genetic correlations, to explore the causality between ADAM/ADAMTS and osteoporosis based on public GWAS data ahead of recruiting new patients or designing additional RCT studies.

The choice of the genetic instrumental variable for MR analysis should fulfil three underlying assumptions including (1) relevance assumption, genetic instrumental variables are reproducibly and strongly associated with the risk factor of interest; (2) independence assumption, genetic variants are not associated with any factors that confound the relationship between exposure and outcome; and (3) exclusion-restriction assumption, instrumental SNPs only influence the outcome through the given exposure [36]. In this study, we selected SNPs illustrating the serum levels of ADAM metallopeptidases based on public GWAS results and analyzed their association with FA-BMD, FN-BMD, LS-BMD and eBMD, since DXA measured BMD has been introduced as diagnostic standard of osteoporosis by world health organization, and meta-analysis of prospective studies have supported the sensitiveness, effectiveness, and independence of quantitative ultrasound estimated heel BMD [37, 38]. Although laboratorial findings reported previously strongly suggested ADAM12, ADAM19, ADAM23, and ADAMTS6 were potential participants in bone metabolism, our two-sample MR estimates had great robustness to support no MR association between levels of these metallopeptidases and osteoporosis based on the results of various MR methods and sensitivity analyses. Meanwhile, the results of our MR study suggested that selected SNPs for higher serum levels of ADAMTS5 were significantly associated with decreased eBMD (*P* < 0.05). Although the significance failed to reach Bonferroni-corrected significance, a robust causal relationship was considered due to the results of additional MR approaches and sensitivity analyses, and the possible linkage disequilibrium among GWAS markers. The power calculation results showed that our study had inadequate power to detect such an association which truly existed but only with a minimal effect, given that variances explained by instrumental variables for ADAMs exposures were relatively small (all < 20%), and sample sizes for BMD outcomes (especially for forearm BMD < 10,000) were largely restricted. It is worth mentioning that, the sample size of GWAS on eBMD was larger than those of GWAS on BMD measured by DXA, which probably accounted for the failure of the association between serum levels of ADAMTS5 and DXA-BMD to reach statistical significance.

In previous studies, ADAMTS5 has always been recognized as a symbolling molecule of osteoarthritis [39, 40], and the positive results in MR analysis suggested its potential role in osteoporosis, which updated the current understanding on this metalloproteinase. A recent study applied ADAMTS5 as one of bone resorption markers, and considered it functional in the process of RAW264.7 cells differentiating into osteoclasts [41], while the underlying mechanism has not been clarified. The relationship between metalloproteinases and bone loss has drawn attention in recent years but remains controversial. Zhu et al. [42] identified a cathepsin K-independent collagenolytic system in osteoclasts, which was composed of a functionally redundant network of the secreted matrix metalloproteinase MMP9 and the membrane-anchored matrix metalloproteinase MMP14. They found that Mmp9/Mmp14 conditional double-knockout mice exhibited marked increases in bone density and displayed a highly protected status against either parathyroid hormone- or ovariectomy-induced pathologic bone loss. Inconsistently, our previous two-sample MR analysis found no evidence for the causal relationship between MMPs and BMD in the European population [43]. Such contradict findings encouraged further explorations in this field. In general, various metalloproteinases are potential in bone loss, especially in bone resorption induced by osteoclasts, and the new evidence for the causal effect of ADAMTS5 on BMD provided by our MR analysis supported further clinical and biological verification.

However, regardless of the strengths, the results of this study should be understood with caution. First, although MR analysis is based on the randomized assignment to the next generation of genes during meiosis, there are still uncontrolled confounding variables since the three key assumptions of the genetic instrumental variable for MR analysis cannot be statistically confirmed. Second, our negative results could not totally deny the potential of ADAM12, ADAM19, ADAM23 and ADAMTS6 as biomarkers for osteoporosis due to the weakness of MR analysis in identifying non-linear relationship [44]. Third, the power and comprehensiveness of MR analysis rely on the availability of GWAS datasets. The genetic data associated with ADAM/ADAMTS used in the MR analysis were based on serum levels, which could not repudiate their physiobiological functions. Given the great pleiotropy of genetic variants and previously intracellular findings, the role of these metallopeptidases in bone metabolism remained worthy of further exploration. Also, all the included participants were of European ancestry, leading to inaccuracy in generalizing our findings to other populations. Besides, ADAM/ADAMTS metalloproteinase family consist of various members, while only five types were analyzed in this study. Previous studies have discovered some types of ADAM/ADAMTS that were not discussed here and might take part in bone remodeling [45]. In addition, it is of great significance, especially in clinical practice, to explore the association of ADAM/ADAMTS with BMD in terms of different age or sex groups, while the GWAS datasets accessible hitherto failed to support further age- or set-stratified analyses. When genetic data of relevance are available in the near future, current analyses could be updated to powerfully provide more informative and comprehensive knowledge on the relationship between ADAM/ADAMTS family and BMD.

## Conclusion

Our study provided suggestive evidence for the causal effect of higher serum levels of ADAMTS5 on decreased heel BMD, while there was no supportive evidence for the associations of ADAM12, ADAM19, ADAM23, and ADAMTS6 with BMD at forearm, femoral neck and lumbar spine in Europeans.

## Electronic supplementary material

Below is the link to the electronic supplementary material.

Supplementary Material 1: Supplementary Tables 1–10

## Data Availability

The original contributions presented in the study are included in the article/Supplementary Material. Data for serum ADAM/ADAMTS levels were gained from SOMAscan platform (https://somalogic.com/somascan-platform/), and summary statistics for BMD used in this study were obtained from the datasets of the largest GWAS on BMD so far released by GEnetic Factors for OSteoporosis Consortium (http://www.gefos.org/?q=content/ukbb-ebmd-gwas-data-release-2017) and the UK Biobank (https://www.ukbiobank.ac.uk/). Further inquiries can be directed to the corresponding author.
