# Supplementary material for: Investigating the association between serum ADAM/ADAMTS levels and bone mineral density by mendelian randomization study
Source: BMC Genomics. 2023 Jul 19;24:406. doi: 10.1186/s12864-023-09449-4 (PMC10354922; doi:10.1186/s12864-023-09449-4)
Supplement: Supplementary file 1 — Supplementary Material 1: Supplementary Tables 1–10 [file 12864_2023_9449_MOESM1_ESM.pdf]

Supplementary Tables

Supplementary Table 1. Data sources of the Mendelian randomization study

Supplementary Table 2. Summary statistics utilized in the Mendelian randomization study of ADAM metallopeptidases on FA-BMD

Supplementary Table 3. Summary statistics utilized in the Mendelian randomization study of ADAM metallopeptidases on FN-BMD

Supplementary Table 4. Summary statistics utilized in the Mendelian randomization study of ADAM metallopeptidases on LS-BMD

Supplementary Table 5. Summary statistics utilized in the Mendelian randomization study of ADAM metallopeptidases on HL-BMD

Supplementary Table 6. Mendelian randomization results for causal effects of ADAM metallopeptidases on FA-BMD

Supplementary Table 7. Mendelian randomization results for causal effects of ADAM metallopeptidases on FN-BMD

Supplementary Table 8. Mendelian randomization results for causal effects of ADAM metallopeptidases on LS-BMD

Supplementary Table 9. Mendelian randomization results for causal effects of ADAM metallopeptidases on HL-BMD

Supplementary Table 10. A *posteriori* power calculations for Mendelian randomization with mRnd

Supplementary Table 1. Data sources of the Mendelian randomization study

Trait	Instrumental SNPs	Sample Size	Ancestry	Adjusted Covariates	Inflation factor λ_{GC}	Author	PMID	Data access Link
ADAM12	2	5,336	European	sex, age, 5 principal components	1.037	Gudjonsson et al.	35078996	https://www.ebi.ac.uk/gwas/studies/GCST90088683
ADAM19	2	5,367	European	sex, age, 5 principal components	1.022	Gudjonsson *et al.*	35078996	https://www.ebi.ac.uk/gwas/studies/GCST90090399
ADAM23	5	5,366	European	sex, age, 5 principal components	1.071	Gudjonsson *et al.*	35078996	https://www.ebi.ac.uk/gwas/studies/GCST90089632
ADAMTS5	3	5,362	European	sex, age, 5 principal components	1.077	Gudjonsson *et al.*	35078996	https://www.ebi.ac.uk/gwas/studies/GCST90088240
ADAMTS6	2	5,357	European	sex, age, 5 principal components	1.055	Gudjonsson *et al.*	35078996	https://www.ebi.ac.uk/gwas/studies/GCST90089427
FA-BMD	–	8,143	European	sex, age, age^2, 5 principal components	1.028	Zheng *et al.*	26367794	https://www.ebi.ac.uk/gwas/studies/GCST005546
FN-BMD	–	32,735	European	sex, age, age^2, 5 principal components	1.062	Zheng *et al.*	26367794	https://www.ebi.ac.uk/gwas/studies/GCST005544
HL-BMD	–	426,824	European	sex, age, age^2, 5 principal components	1.060	Morris *et al.*	30598549	https://www.ebi.ac.uk/gwas/studies/GCST006979
LS-BMD	–	28,498	European	sex, age, age^2, 5 principal components	1.065	Zheng *et al.*	26367794	https://www.ebi.ac.uk/gwas/studies/GCST005545

Abbreviations: BMD, bone mineral density; FA, forearm; FN, femoral neck; HL, heel; LS, lumbar spine; SNP, single nucleotide polymorphism.

Supplementary Table 2. Summary statistics utilized in the Mendelian randomization study of ADAM metallopeptidases on FA-BMD

ADAM family	SNP	Position	EA/OA	Association with ADAMs				Association with FA-BMD		
				Beta	SE	*P*-value	*F*-statistic	Beta	SE	*P*-value
ADAM12	rs11244755	10:127687017	T/C	0.186	0.019	9.90×10^{-23}	95.8	0.010	0.017	0.581
ADAM19	rs7728609	5:156935524	T/C	-0.238	0.020	1.10×10^{-33}	141.6	0.006	0.017	0.728
ADAM19	rs185289117	14:106017813	G/A	0.630	0.061	3.50×10^{-25}	106.7	0.079	0.087	0.371
ADAM23	rs1448903	2:207308961	G/A	0.517	0.031	7.70×10^{-63}	278.1	-0.045	0.027	0.102
ADAM23	rs143219430	2:207392258	C/A	-0.817	0.057	8.40×10^{-47}	205.4	-0.081	0.052	0.128
ADAM23	rs7598482	2:207340139	T/C	-0.253	0.019	1.10×10^{-38}	177.3	0.002	0.016	0.904
ADAM23	rs12471404	2:207509762	C/A	-0.641	0.056	3.40×10^{-30}	131.0	0.009	0.049	0.852
ADAM23	rs11045856	12:21350689	G/T	-0.173	0.022	7.40×10^{-15}	61.8	-0.011	0.019	0.559
ADAMTS5	rs2830585	21:28305212	T/C	-1.073	0.044	4.00×10^{-104}	601.2	-0.039	0.021	0.070
ADAMTS5	rs990529	21:28346546	G/C	0.329	0.041	1.59×10^{-15}	65.6	0.028	0.015	0.081
ADAMTS5	rs229100	21:28223092	T/C	0.366	0.052	2.87×10^{-12}	50.0	0.029	0.021	0.169
ADAMTS6	rs75575009	11:18220689	C/T	0.699	0.054	1.00×10^{-37}	167.6	0.059	0.054	0.278
ADAMTS6	rs704	17:26694861	A/G	0.196	0.018	2.80×10^{-28}	118.6	-0.002	0.020	0.910

Abbreviations: ADAM, A Disintegrin And Metalloproteinase; ADAMTS, ADAM With Thrombospondin Motifs; BMD, bone mineral density; FA, forearm; SNP, single nucleotide polym[illegible]

Supplementary Table 3. Summary statistics utilized in the Mendelian randomization study of ADAM metallopeptidases on FN-BMD

ADAM family	SNP	Position	EA/OA	Association with ADAMs				Association with FN-BMD		
				Beta	SE	*P*-value	*F*-statistic	Beta	SE	*P*-value
ADAM12	rs11244755	10:127687017	T/C	0.186	0.019	9.90×10^{-23}	95.8	-0.009	0.008	0.272
ADAM19	rs7728609	5:156935524	T/C	-0.238	0.020	1.10×10^{-33}	141.6	0.000	0.008	0.953
ADAM19	rs185289117	14:106017813	G/A	0.630	0.061	3.50×10^{-25}	106.7	0.026	0.044	0.565
ADAM23	rs1448903*	2:207311349	G/A	0.517	0.031	7.70×10^{-63}	278.1	-0.027	0.012	0.032
ADAM23	rs143219430	2:207392258	C/A	-0.817	0.057	8.40×10^{-47}	205.4	-0.023	0.026	0.377
ADAM23	rs7598482	2:207340139	T/C	-0.253	0.019	1.10×10^{-38}	177.3	-0.015	0.008	0.069
ADAM23	rs12471404	2:207509762	C/A	-0.641	0.056	3.40×10^{-30}	131.0	0.012	0.024	0.614
ADAM23	rs11045856	12:21350689	G/T	-0.173	0.022	7.40×10^{-15}	61.8	-0.015	0.009	0.103
ADAMTS5	rs2830585	21:28329010	T/C	-1.073	0.044	4.00×10^{-104}	601.2	0.004	0.010	0.678
ADAMTS5	rs990529	21:28346546	G/C	0.329	0.041	1.59×10^{-15}	65.6	-0.010	0.008	0.197
ADAMTS5	rs229100	21:28223092	T/C	0.366	0.052	2.87×10^{-12}	50.0	-0.020	0.010	0.051
ADAMTS6	rs75575009	11:18220689	C/T	0.699	0.054	1.00×10^{-37}	167.6	0.000	0.026	0.987

* For rs1448903 and rs2830585 which were not present in in the summary statistics of FN-BMD, rs72933203 (chr2:207311349, r2 = 0.91) and rs233599 (chr21:28329010, r2 = 0.90) were utilized as proxies, respectiv

Abbreviations: ADAM, A Disintegrin And Metalloproteinase; ADAMTS, ADAM With Thrombospondin Motifs; BMD, bone mineral density; FN, femoral neck; SNP, single nucleotide polymorphism.

Supplementary Table 4. Summary statistics utilized in the Mendelian randomization study of ADAM metallopeptidases on LS-BMD

ADAM family	SNP	Position	EA/OA	Association with ADAMs				Association with LS-BMD		
				Beta	SE	*P*-value	*F*-statistic	Beta	SE	*P*-value
ADAM12	rs11244755	10:127687017	T/C	0.186	0.019	9.90×10^{-23}	95.8	0.004	0.009	0.689
ADAM19	rs7728609	5:156935524	T/C	-0.238	0.020	1.10×10^{-33}	141.6	0.002	0.009	0.806
ADAM19	rs185289117	14:106017813	G/A	0.630	0.061	3.50×10^{-25}	106.7	0.073	0.051	0.160
ADAM23	rs1448903	2:207319653	G/A	0.517	0.031	7.70×10^{-63}	278.1	-0.011	0.014	0.466
ADAM23	rs143219430	2:207392258	C/A	-0.817	0.057	8.40×10^{-47}	205.4	0.030	0.030	0.328
ADAM23	rs7598482	2:207340139	T/C	-0.253	0.019	1.10×10^{-38}	177.3	0.012	0.009	0.215
ADAM23	rs12471404	2:207509762	C/A	-0.641	0.056	3.40×10^{-30}	131.0	-0.022	0.028	0.438
ADAM23	rs11045856	12:21350689	G/T	-0.173	0.022	7.40×10^{-15}	61.8	-0.028	0.010	0.007
ADAMTS5	rs2830585	21:28329010	T/C	-1.073	0.044	4.00×10^{-104}	601.2	0.004	0.012	0.762
ADAMTS5	rs990529	21:28346546	G/C	0.329	0.041	1.59×10^{-15}	65.6	-0.005	0.009	0.610
ADAMTS5	rs229100	21:28223092	T/C	0.366	0.052	2.87×10^{-12}	50.0	0.022	0.012	0.063
ADAMTS6	rs75575009	11:18220689	C/T	0.699	0.054	1.00×10^{-37}	167.6	-0.004	0.030	0.902

* For rs1448903 and rs2830585 which were not present in in the summary statistics of LS-BMD, rs72933203 (chr2:207311349, r2 = 0.91) and rs233599 (chr21:28329010, r2 = 0.90) were utilized as proxies, respective
Abbreviations: ADAM, A Disintegrin And Metalloproteinase; ADAMTS, ADAM With Thrombospondin Motifs; BMD, bone mineral density; LS, lumbar spine; SNP, single nucleotide polymorphism.

Supplementary Table 5. Summary statistics utilized in the Mendelian randomization study of ADAM metallopeptidases on HL-BMD

ADAM family	SNP	Position	EA/OA	Association with ADAMs				Association with HL-BMD		
				Beta	SE	*P*-value	*F*-statistic	Beta	SE	*P*-value
ADAM12	rs11244755	10:127687017	T/C	0.186	0.019	9.90×10^{-23}	95.8	0.003	0.002	0.750
ADAM12	rs35214304	10:127730298	G/T	0.153	0.028	4.00×10^{-8}	29.9	-0.014	0.003	0.001
ADAM19	rs7728609	5:156935524	T/C	-0.238	0.020	1.10×10^{-33}	141.6	-0.003	0.002	0.190
ADAM19	rs185289117	14:106017813	G/A	0.630	0.061	3.50×10^{-25}	106.7	0.007	0.010	0.120
ADAM23	rs1448903	2:207308961	G/A	0.517	0.031	7.70×10^{-63}	278.1	-0.004	0.003	0.210
ADAM23	rs143219430	2:207392258	C/A	-0.817	0.057	8.40×10^{-47}	205.4	0.013	0.006	0.087
ADAM23	rs7598482	2:207340139	T/C	-0.253	0.019	1.10×10^{-38}	177.3	0.003	0.002	0.130
ADAM23	rs12471404	2:207509762	C/A	-0.641	0.056	3.40×10^{-30}	131.0	0.001	0.006	0.900
ADAM23	rs11045856	12:21350689	G/T	-0.173	0.022	7.40×10^{-15}	61.8	-0.011	0.002	0.001
ADAMTS5	rs2830585	21:28305212	T/C	-1.073	0.044	4.00×10^{-104}	601.2	0.006	0.003	0.099
ADAMTS5	rs990529	21:28346546	G/C	0.329	0.041	1.59×10^{-15}	65.6	-0.003	0.002	0.240
ADAMTS5	rs229100	21:28223092	T/C	0.366	0.052	2.87×10^{-12}	50.0	-0.001	0.003	0.740
ADAMTS6	rs75575009	11:18220689	C/T	0.699	0.054	1.00×10^{-37}	167.6	-0.009	0.006	0.110
ADAMTS6	rs704	17:26694861	A/G	0.196	0.018	2.80×10^{-28}	118.6	-0.016	0.002	0.001

Abbreviations: ADAM, A Disintegrin And Metalloproteinase; ADAMTS, ADAM With Thrombospondin Motifs; BMD, bone mineral density; HL, heel; SNP, single nucleotide polymorphism.

Supplementary Table 6. Mendelian randomization results for causal effects of ADAM metallopeptidases on FA-BMD

Traits	No. of SNPs	Effect (95% CI)	*P*-value	Heterogeneity I^2	*P*-value	MR-Egger intercept	*P*-value
**ADAM12**							
Inverse-variance weighted	1	0.051 (-0.127, 0.230)	0.573	NA	NA		
Weighted median							
MR-Egger regression						NA	NA
**ADAM19**							
Inverse-variance weighted	2	0.006 (-0.116, 0.127)	0.925	0.0%	0.330		
Weighted median							
MR-Egger regression						NA	NA
**ADAM23**							
Inverse-variance weighted	5	-0.006 (-0.075, 0.063)	0.866	88.0%	0.001		
Weighted median	5	-0.011 (-0.088, 0.065)	0.772				
MR-Egger regression	5	-0.011 (-0.175, 0.152)	0.900			0.002	0.945
**ADAMTS5**							
Inverse-variance weighted	2	0.031 (-0.014, 0.077)	0.277	0.00%	0.799		
Weighted median							
MR-Egger regression						NA	NA
**ADAMTS6**							
Inverse-variance weighted	2	0.051 (-0.070, 0.171)	0.412	96.5%	0.001		
Weighted median							
MR-Egger regression						NA	NA

Note: Weighted median and MR-Egger regression methods were available only when there were required instrumental variables (n ≥ 3). Inverse-variance weighted estimate was equal to Wald ratio-based estimate when there was one instrumental variable alone.

Abbreviations: ADAM, A Disintegrin And Metalloproteinase; ADAMTS, ADAM With Thrombospondin Motifs; BMD, bone mineral density; FA, forearm; SNP, single nucleotide polymorphis

Supplementary Table 7. Mendelian randomization results for causal effects of ADAM metallopeptidases on FN-BMD

Traits	No. of SNPs	Effect (95% CI)	*P*-value	Heterogeneity I^2	*P*-value	MR-Egger intercept	*P*-value
**ADAM12**							
Inverse-variance weighted	1	-0.049 (-0.134, 0.036)	0.261	NA	NA		
Weighted median							
MR-Egger regression						NA	NA
**ADAM19**							
Inverse-variance weighted	2	0.009 (-0.050, 0.068)	0.759	0.00%	0.613		
Weighted median							
MR-Egger regression						NA	NA
**ADAM23**							
Inverse-variance weighted	5	0.003 (-0.045, 0.052)	0.892	66.8%	0.017		
Weighted median	5	0.000 (-0.040, 0.040)	0.999				
MR-Egger regression	5	-0.056 (-0.142, 0.029)	0.288			0.024	0.216
**ADAMTS5**							
Inverse-variance weighted	2	-0.010 (-0.041, 0.021)	0.542	66.9%	0.082		
Weighted median							
MR-Egger regression						NA	NA
**ADAMTS6**							
Inverse-variance weighted	1	0.001 (-0.072, 0.073)	0.987	NA	NA		
Weighted median							
MR-Egger regression						NA	NA

Note: Weighted median and MR-Egger regression methods were available only when there were required instrumental variables (n ≥ 3). Inverse-variance weighted estimate was equal to Wald ratio-based estimate when there was one instrumental variable alone.

Abbreviations: ADAM, A Disintegrin And Metalloproteinase; ADAMTS, ADAM With Thrombospondin Motifs; BMD, bone mineral density; FA, forearm; SNP, single nucleotide polymorphis

Supplementary Table 8. Mendelian randomization results for causal effects of ADAM metallopeptidases on LS-BMD

Traits	No. of SNPs	Effect (95% CI)	*P*-value	Heterogeneity I^2	*P*-value	MR-Egger intercept	*P*-value
**ADAM12**							
Inverse-variance weighted	1	0.021 (-0.078, 0.120)	0.682	NA	NA		
Weighted median							
MR-Egger regression						NA	NA
**ADAM19**							
Inverse-variance weighted	2	0.014 (-0.082, 0.110)	0.777	49.4%	0.160		
Weighted median							
MR-Egger regression						NA	NA
**ADAM23**							
Inverse-variance weighted	5	-0.007 (-0.062, 0.048)	0.809	64.7%	0.023		
Weighted median	5	-0.027 (-0.067, 0.014)	0.194				
MR-Egger regression	5	-0.056 (-0.170, 0.058)	0.408			0.020	0.406
**ADAMTS5**							
Inverse-variance weighted	2	0.004 (-0.036, 0.043)	0.858	72.2%	0.058		
Weighted median							
MR-Egger regression						NA	NA
**ADAMTS6**							
Inverse-variance weighted	1	-0.005 (-0.089, 0.078)	0.899	NA	NA		
Weighted median							
MR-Egger regression						NA	NA

Note: Weighted median and MR-Egger regression methods were available only when there were required instrumental variables (n ≥ 3). Inverse-variance weighted estimate was equal to Wald ratio-based estimate when there was one instrumental variable alone.

Abbreviations: ADAM, A Disintegrin And Metalloproteinase; ADAMTS, ADAM With Thrombospondin Motifs; BMD, bone mineral density; LS, lumbar spine; SNP, single nucleotide polymorphism.

Supplementary Table 9. Mendelian randomization results for causal effects of ADAM metallopeptidases on HL-BMD

Traits	No. of SNPs	Effect (95% CI)	*P*-value	Heterogeneity I^2	*P*-value	MR-Egger intercept	*P*-value
**ADAM12**							
Inverse-variance weighted	2	-0.008 (-0.092, 0.075)	0.847	95.1%	0.001		
Weighted median							
MR-Egger regression						NA	NA
**ADAM19**							
Inverse-variance weighted	2	0.014 (0.000, 0.028)	0.055	0.00%	0.880		
Weighted median							
MR-Egger regression						NA	NA
**ADAM23**							
Inverse-variance weighted	5	-0.004 (-0.024, 0.016)	0.684	27.9%	0.235		
Weighted median	5	-0.011 (-0.019, -0.002)	0.019				
MR-Egger regression	5	-0.027 (-0.063, 0.010)	0.244			0.009	0.255
**ADAMTS5**							
Inverse-variance weighted	3	-0.006 (-0.010, -0.002)	**0.004**	0.00%	0.467		
Weighted median	3	-0.006 (-0.010, -0.002)	**0.006**				
MR-Egger regression	3	-0.005 (-0.013, 0.003)	0.411			-0.001	0.874
**ADAMTS6**							
Inverse-variance weighted	2	-0.047 (-0.116, 0.022)	0.185	0.00%	0.452		
Weighted median							
MR-Egger regression						NA	NA

Note: Weighted median and MR-Egger regression methods were available only when there were required instrumental variables (n ≥ 3). Inverse-variance weighted estimate was equal to Wald ratio-based estimate when there was one instrumental variable alone.

Abbreviations: ADAM, A Disintegrin And Metalloproteinase; ADAMTS, ADAM With Thrombospondin Motifs; BMD, bone mineral density; HL, heel; SNP, single nucleotide polymorphism.

Supplementary Table 10. A *posteriori* power calculations for Mendelian randomization with mRnd

Exposures	Outcomes	Sample size for outcome	Variance explained for exposure	Estimate for causal effect	Power calculation by mRnd
**ADAM12**	FA-BMD	8143	0.023	0.051	0.109
	FN-BMD	32735	0.023	-0.049	0.270
	HL-BMD	426824	0.023	0.021	0.541
	LS-BMD	28498	0.023	-0.008	0.055
**ADAM19**	FA-BMD	8143	0.046	0.006	0.051
	FN-BMD	32735	0.046	0.009	0.065
	HL-BMD	426824	0.046	0.014	0.496
	LS-BMD	28498	0.046	0.014	0.080
**ADAM23**	FA-BMD	8143	0.152	-0.006	0.055
	FN-BMD	32735	0.152	0.003	0.057
	HL-BMD	426824	0.152	-0.007	0.408
	LS-BMD	28498	0.152	-0.004	0.058
**ADAMTS5**	FA-BMD	8143	0.122	0.031	0.165
	FN-BMD	32735	0.122	-0.010	0.094
	HL-BMD	426824	0.122	0.004	0.131
	LS-BMD	28498	0.122	-0.006	0.065
**ADAMTS6**	FA-BMD	8143	0.052	0.051	0.180
	FN-BMD	32735	0.052	0.001	0.050
	HL-BMD	426824	0.052	-0.005	0.126
	LS-BMD	28498	0.052	-0.047	0.440

Note: https://shiny.cnsgenomics.com/mRnd/

Abbreviations: ADAM, A Disintegrin And Metalloproteinase; ADAMTS, ADAM With Thrombospondin Motifs; BMD, bone mineral density; FA, forearm; SNP, single nucleotide polymorphism.
